# Low-frequency electrotherapy for female patients with detrusor underactivity due to neuromuscular deficiency

**DOI:** 10.1007/s00192-012-1714-2

**Published:** 2012-03-23

**Authors:** Dan-Feng Xu, Shen Zhang, Cun-Zhou Wang, Jun Li, Chuang-Yu Qu, Xin-Gang Cui, Sheng-Jia Zhao

**Affiliations:** 1 Department of Urology, Changzheng Hospital, Second Military Medical University, No. 415, Fengyang Rd, Shanghai, 200003 China; 2 Department of Physiotherapy, Changzheng Hospital, Second Military Medical University, Shanghai, China

**Keywords:** Detrusor underactivity, Electrotherapy, Functional stimulation, Low frequency, Lower urinary tract dysfunction, Urodynamics

## Abstract

**Introduction and hypothesis:**

The aim of the study was to assess the efficacy of low-frequency electrotherapy (LFE) for female patients with early-stage detrusor underactivity (DUA) due to neuromuscular deficiency.

**Methods:**

A total of 102 female patients were divided randomly into four groups: LFE-NC (normal compliance), LFE-LC (low compliance), CON (control)-NC and CON-LC. Patients in the LFE-NC and LFE-LC groups received LFE, and those in the CON-NC and CON-LC groups received conservative treatment. Urodynamic evaluation was performed before and after treatment.

**Results:**

After treatment, 82 % of the LFE-NC regained detrusor contractility, whereas only 2 (8 %) of the CON-NC had normal detrusor contraction. None of LFE-LC or CON-LC regained detrusor contractility (*p* < 0.01). The per cent of LFE-NC who relied on catheterization for bladder emptying decreased by 43 % (*p* < 0.01). Those in the LFE-LC, CON-NC and CON-LC groups decreased by only 4, 12 or 0 % (*p* > 0.05).

**Conclusions:**

LFE was more effective for DUA patients with normal compliance; these patients benefited from LFE, but DUA patients with low compliance did not.

## Introduction

The detrusor and sphincter are two key functional elements of the lower urinary tract. Either detrusor or sphincter overactivity or underactivity will lead to abnormal voiding conditions [1, 2]. Normal voiding implies the ability to quickly and completely empty a bladder recognized as being normally full. Detrusor underactivity (DUA) or underactive bladder (UAB) is a diagnosis made on a pressure-flow study (PFS). Such a condition is characterized by a low sustained or wavelike contraction and is associated with poor flow [3, 4]. The International Continence Society (ICS) defines DUA as “a detrusor contraction of inadequate magnitude and/or duration to effect complete bladder emptying in the absence of urethral obstruction” [5, 6]. Failure to accomplish any aspect of this triad (recognition, speed, efficiency) are the hallmarks of impaired voiding function. In this context “recognition” means understanding or being aware of the significance of impaired voiding function, “speed” implies fast reaction to the dysfunction and “efficiency” denotes full response to the management. DUA may be a manifestation of aging too [7]. DUA or incomplete bladder emptying is a risk factor for urinary tract infection, developing a high intravesical pressure and large quantity of residual urine volume and overflow incontinence[3]. Methods to improve the voiding process are practised in patients with DUA and other lower urinary tract dysfunction (LUTD), such as: third party bladder expression (Credé’s manoeuvre), voiding by abdominal straining (Valsalva’s manoeuvre) and triggered reflex voiding, indwelling or intermittent self-catheterization (ISC) [1] or some kind of remote-controlled intraurethral device [8]. Indwelling catheters have the ongoing risk of infection and over time many women develop a patulous urethra, which becomes a major management issue. The management option for DUA is limited. To date, there is no effective, outcome-validated drug available for DUA [3, 9].

Patients with an acute episode often have an urgent desire to manipulate their DUA. Such conditions may result from iatrogenic factors (overdistention of the bladder after operation, neurogenic injury associated with abdominoperineal resection of rectum, simple or radical hysterectomy or even pelvic irradiation for cervical cancer) [1] or occur with complaints of acute urinary retention on account of some individual misguided behaviour or with incidentally discovered lumbosacral abnormality, such as intervertebral tube stenosis or intervertebral disk hernia. DUA patients were further subdivided into normal compliance (NC) and low compliance (LC), observed during the filling phase [5].

Low-frequency electrotherapy (LFE) is a noninvasive approach of delivering surface electrical stimulation, representing a functional stimulation therapy. The LFE unit has been used in this institution since 2008. To further assess the utility of this relatively novel treatment in women with DUA refractory to behavioural and pharmacotherapy, we studied the outcome of LFE in the current patient population.

## Methods

### Clinical experimental design

**Fig. 1:**
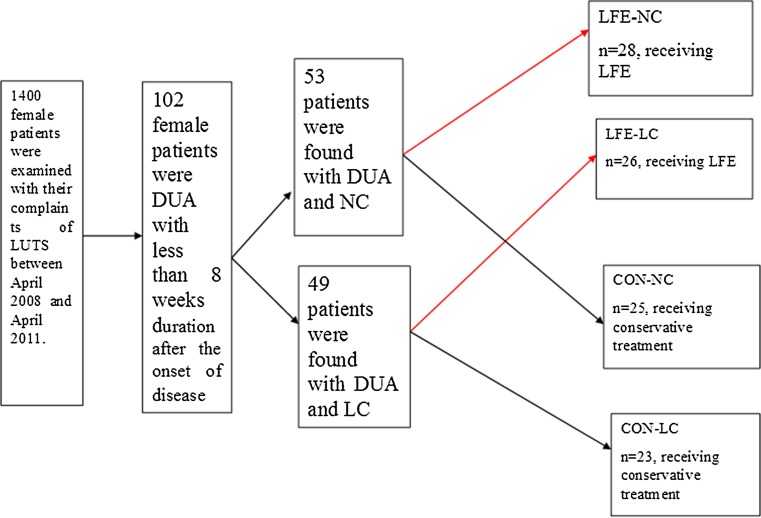
Flow diagram of the experimental design and the patient grouping

Of the 1,400 female patients examined with complaints of lower urinary tract symptoms (LUTS) between April 2008 and April 2011 in this institution, 102 women with symptoms (poor/weak flow, frequency or even urinary rentention) and urodynamic diagnosis of DUA (with a finding of inability to initiate a detrusor contraction or detrusor contraction curve in a sustained low-level or wavelike fashion) were enrolled (Fig. 1). Of these DUA patients, 53 had DUA-NC and 49 had DUA-LC. Patients were allocated into four groups according to their compliance and treatment desire: LFE-NC (*n* = 28, DUA-NC receiving LFE), LFE-LC (*n* = 26, DUA-LC receiving LFE), CON-NC (control, with conservative treatment, *n* = 25, DUA-NC not receiving LFE) or CON-LC (*n* = 23, DUA-LC not receiving LFE). Patients in the LFE-NC and LFE-LC groups received two treatment sessions (each lasting for 70 min) daily and routine conservative treatment for 2 weeks. Patients in the CON-NC and CON-LC groups received routine conservative treatment (Fig. 1).

LFE using ElectroStimulation 420 (ES-420, Ito Co., Ltd., Tokyo, Japan) was not conducted in the physiotherapy department of this institution until April 2008. Prior to this study, patients with symptomatic DUA were treated routinely with pyridostigmine bromide, baclofen, Chinese medicinal herb soup and ISC. With the availability of ES-420, a small group of patients with symptomatic DUA was subjected to LFE. A retrospective comparative trial was initiated to assess the treatment method and outcomes.

The LFE unit for the study was provided by Ito Co., Ltd., Tokyo, Japan, which had no role in the design or implementation of the study, the analysis or interpretation of the data, or the preparation of the manuscript. LFE was employed to stimulate cells in the body that respond to electrical charge, such as muscle and nerve cells. Weak direct currents that move in a pulsating rhythm are delivered to the affected organ or area.

The main provocative or associated causes of DUA were iatrogenic factors (overdistention of the bladder following surgery of the liver, intestine or spleen) or individual misguided behaviour (abrupt voluntary withholding of urination or voiding in an unnatural position, i.e. with her haunch raised up to avoid touching the commode), nerve injury associated with operation for malignancy of the colon, rectum or uterus, muscular deficiency due to dystocia or prolonged labour delivery, and intervertebral disk hernia or vertebral tube stenosis. In some patients, no obvious origin was found (Table 1).

**Table 1:** Distribution of provocative or associated causes of detrusor underactivity at presentation, *n* (%)

Cause analysis	DUA with normal compliance (*n* = 53)	DUA with low compliance (*n* = 49)
Bladder overdistention due to iatrogenic origin or prolonged delivery	10 (19)	9 (18)
Nerve injury associated with operation for diseases of		
The colon or rectum	15 (28)	16 (33)
The uterus	13 (25)	13 (27)
The vertebral column	4 (7)	5 (10)
Unknown cause	11 (21)	6 (12)

### Inclusion and exclusion criteria

The local Ethics Committee granted its approval and all participants provided written informed consents. Patients with an initial acute condition and a subtle neurogenic origin with a short duration (less than 8 weeks) after the onset of disease were included, whereas those with obvious chronic neurological injury or disease with a long duration after the onset of disease (more than 8 weeks), such as spinal cord injury, stroke or spinal tumours, were excluded from the study. The exclusion criteria also included pregnancy and structural abnormalities of the lower urinary tract.

### Routine evaluation

All patients with LUTD receiving urodynamic evaluation underwent physical examination, comprehensive history taking and urinalysis. The standard urological evaluation included pelvic and neurourological examination, with determination of pelvic floor muscle strength, assessment of reflexes and perineal-perianal sensation. Further evaluation included ultrasonography of the urinary tract and excretory urography if there was persistent haematuria.

### Urodynamic evaluation

The urodynamic investigations (Urovision Janus System V, Life Tech, Inc., Stafford, TX, USA) included maximum flow rate (free Q_max_), filling cystometrography (CMG), voiding pressure-flow study (PFS), external anal sphincter electromyography (EAS EMG) and urethral pressure profilometry (UPP) according to previously described techniques [10, 11]. Methods, definitions and units were employed according to the standards recommended by the ICS, except where specifically noted [5, 6]. During CMG and PFS, vesical pressure (P_ves_) and abdominal pressure (P_abd_) and then detrusor pressure (P_det_ = P_ves_-P_abd_) were measured by using a two-lumen 8 French catheter and a rectal balloon catheter, respectively. EAS EMG was also simultaneously monitored using two needle-guided wire electrodes inserted at 3 and 9 o’clock of the anus aperture with a lateral distance of 0.5 cm. Filling CMG was performed with a rate of 50–70 ml/min of saline and the compliance was recorded continuously. The infusion was stopped to initiate voiding with changing position from supine to sitting when maximum cystometric capacity (MCC) was reached. The urinary flow rates, P_det_ and detrusor contraction fashion were measured during the voiding phase when the patient was instructed to void. Finally, the UPP was obtained and the maximum urethral closure pressure (MUCP) and the functional profile length (FPL) were recorded.

DUA was defined as a detrusor contraction of inadequate magnitude and/or duration or even no contraction at all to effective and complete bladder emptying in the absence of urethral obstruction [5]. Normal compliance was defined as more than 15 ml/cmH_2_O [1, 5, 12].

Sphincter overactivity was diagnosed when there was increased external sphincter activity during voluntary voiding, as shown by the increased sphincter activity with a sustained detrusor contraction during PFS [5]. Sphincter overactivity degree was expressed with a quantitative analysis of the potentials using the parameter of tense/loose (TL) value as previously described [10, 11, 22]. Briefly, it was derived from EAS EMG and was expressed as [lg (potentials before voiding/at Q_max_)] with a negative number (<0) indicating that the sphincter activity was abnormally increased during voiding. The detrusor contraction fashion in DUA-NC was low sustained (Fig. 3a), whereas those in DUA-LC was high sustained as the P_det_ had reached a relatively high level prior to ordering voiding and did not increase during the voiding phase (Fig. 4a) [12, 13]. If effective detrusor contraction pressure was claimed, the P_det_ ahead of the voiding order should be subtracted from the real-time P_det_.

The comprehensive urodynamic study was repeated at least twice to obtain a reproducible PFS tracing, especially P_abd_ and P_det_, to show the ability of the detrusor to contract or the abdominal strain to compensate for failure of bladder contraction (Fig. 4c, d). The first study was conducted prior to the initiation of LFE as the baseline, and the follow-up examination was conducted at least 4 weeks after the conclusion of LFE.

### Therapeutic programme

**Fig. 2:**
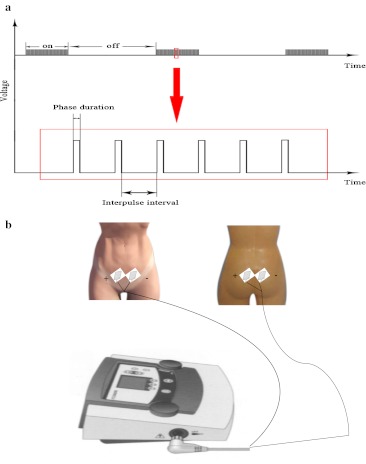
Diagrams for LFE method. Diagram of monophasic pulsed rectangular direct current showing phase duration, interpose interval and on/off of the current (**a**), and diagram of electrode placement from the anterior and posterior views of a female patient (**b**)

The patient was lying on the table. The indwelling catheter was left in place and the connecting tube was not clamped, so that the bladder was maintained in the empty state during the procedure. The two rectangular 50 × 90 mm self-adhesive electrode plates were applied to the skin overlying the skin of the second sacral foramina and another two plates applied to the skin 3.0 cm under the umbilicus on the abdomen. The electrode plates were connected to the electrical stimulator LFE unit via leads(Fig. 2b). This procedure ensures accuracy and maintains a standardized electrode placement between sessions and for all participants. At first, the preset programme A (f) of the electrical muscle stimulation was used for 20 min. The generator delivered monophasic pulsed direct current (20 Hz with phase duration 5 s, wave width 150 μs and on/off time 5 s/10 s) through the bladder detrusor and the adjacent tissue, followed by programme A (a) for the next 20 min (monophasic pulsed direct current of 80 Hz with phase duration 10 s, wave width 300 μs and on/off time 10 s/50 s) (Fig. 2a). The stimulation intensity was increased by the instructor as long as the participant felt the sensation was strong and comfortable, but never above the motor threshold. If a motor response was detected, the intensity was reduced to eliminate any muscle contraction.

Each patient of the LFE-NC or LFE-LC groups was subjected to two treatment sessions each day for a total of 2 weeks, meanwhile with conservative treatment. Patients of the CON-NC or CON-LC groups received conservative treatment as those before LFE was conducted. The primary outcome measurement was defined as the changes of the detrusor contractility status and indwelling catheter, whether it was still required or could be abandoned, from the baseline to the follow-up assessment. The secondary outcome measurements included other urodynamic variables, especially the compliance and PFS data.

### Data collection and statistical analysis

All statistical analyses were carried out with Statistical Package for the Social Sciences software (SPSS 10, Chicago, IL, USA). The values and variables were indicated as mean ± standard deviation. The *t* test was performed for comparison of the difference of two groups. The chi-square test was used to evaluate the categorical variables, and the McNemar test was used to compare the follow-up data. Differences and correlations were considered as statistically significant at *p* < 0.05.

## Results

There was no significant difference in the distribution of provocative or associated causes of DUA at presentation between DUA with NC or with LC (Table 1).There were no significant differences in parameters such as age, duration after the onset of disease, frequency or nocturia at the baseline between the subgroups with NC or LC in either LFE or CON groups (*p* > 0.05, Table 2). Patients of the LFE-NC and LFE-LC groups all completed their LFE therapy and had their urodynamic evaluation repeated according to the protocol (Table 2). The TL values of the patients of the LFE-NC group increased at follow-up as compared with those at baseline (0.75 ± 0.06 vs −0.91 ± 0.07, *p* < 0.01) and those of other groups (LFE-LC, CON-NC and CON-LC, *p* < 0.01). There were no significant differences between baseline and follow-up in MCC, compliance, MUCP and FPL (*p* > 0.05).

**Table 2:** Symptomatic and urodynamic data of 102 women with detrusor underactivity administered conservative treatment or low-frequency electrotherapy for 2 weeks

Variables	LFE (*n* = 54)		Controls (*n* = 48)	
	LFE-NC (*n* = 28)	LFE-LC (*n* = 26)	CON-NC (*n* = 25)	CON-LC (*n* = 23)
Mean (SD)				
Age, years	53.6 (2.1)	55.6 (2.3)	56.2 (4.1)	54.5 (4.0)
Duration after the onset of disease, weeks	5.5 (1.2)	5.0 (1.1)	4.1 (1.0)	4.1 (1.0)
Frequency, no. of voids/24 h	13.3 (3.5)	14.3 (3.4)	12.3 (2.4)	12.6 (3.2)
Nocturia, no. of voids	4.5 (2.2)	5.1 (2.1)	5.4 (1.7)	5.5 (1.4)
At baseline				
MCC, ml	353 (140)**	160 (38)	346 (105)**	105 (46)
Compliance, ml/cmH_2_O	36.5 (10.5)**	13.0 (6.6)	41.2 (12.5)**	12.7 (5.5)
TL value	−0.91 (0.07)	−0.93 (0.07)	–0.96 (0.10)	−0.92 (0.12)
MUCP, cmH_2_O	52.5 (14.7)	53.4 (10.3)	45.9 (12.3)	50.1 (19.5)
FPL, cm	2.6 (1.0)	2.8 (0.9)	2.9 (0.6)	3.0 (0.7)
Follow-up after 4 weeks				
MCC, ml	340 (110)**	154 (35)	364 (109)**	95 (40)
Compliance, ml/cmH_2_O	40.5 (12.5)**	15.0 (7.6)	40.2 (10.5)**	13.2 (4.5)
TL value	0.75 (0.06)*, **	−0.91 (0.09)	−0.96 (0.10)	−0.92 (0.12)
MUCP, cmH_2_O	45.1 (12.2)	48.4 (10.5)	45.1 (13.5)	50.5 (10.7)
FPL, cm	2.7 (0.8)	2.9 (0.8)	2.8 (0.7)	3.1 (0.4)
*n* (%)				
At baseline				
Relied on catheterization for bladder emptying	15 (54)	22 (85)	15 (60)	20 (87)
Relied on abdominal straining for bladder emptying	13 (46)	4 (15)	10 (40)	3 (13)
Detrusor contraction fashion				
Low or high sustained	9 (32)	20 (77)	8 (32)	18 (78)
Wavelike	19 (68)	6 (23)	17 (68)	5 (22)
Parabola	0 (0)	0 (0)	0 (0)	0 (0)
Follow-up after 4 weeks				
Relied on catheterization for bladder emptying	3 (11)	21 (81)	12 (48)	20 (87)
Relied on abdominal straining for bladder emptying	2 (7)	5 (19)	11 (44)	3 (13)
Returning to normal	23 (82)*, **	0 (0)	2 (8)	0 (0)
Detrusor contraction fashion				
Low or high sustained	1 (4)	22 (84)	8 (32)	20 (87)
Wavelike	3 (10)	4 (16)	16 (64)	3 (13)
Parabola	24 (86)*, **	0 (0)	1 (4)	0 (0)

*DUA* detrusor underactivity, *FPL* functional profile length, *LC* low compliance, *LFE* low-frequency electrotherapy, *MCC* maximal cystometric capacity, *MUCP* maximum urethral closure pressure, *NC* normal compliance

*p < 0.01, as compared with that before LFE; **p < 0.01, data with NC as compared with those with LC

**Fig. 3:**
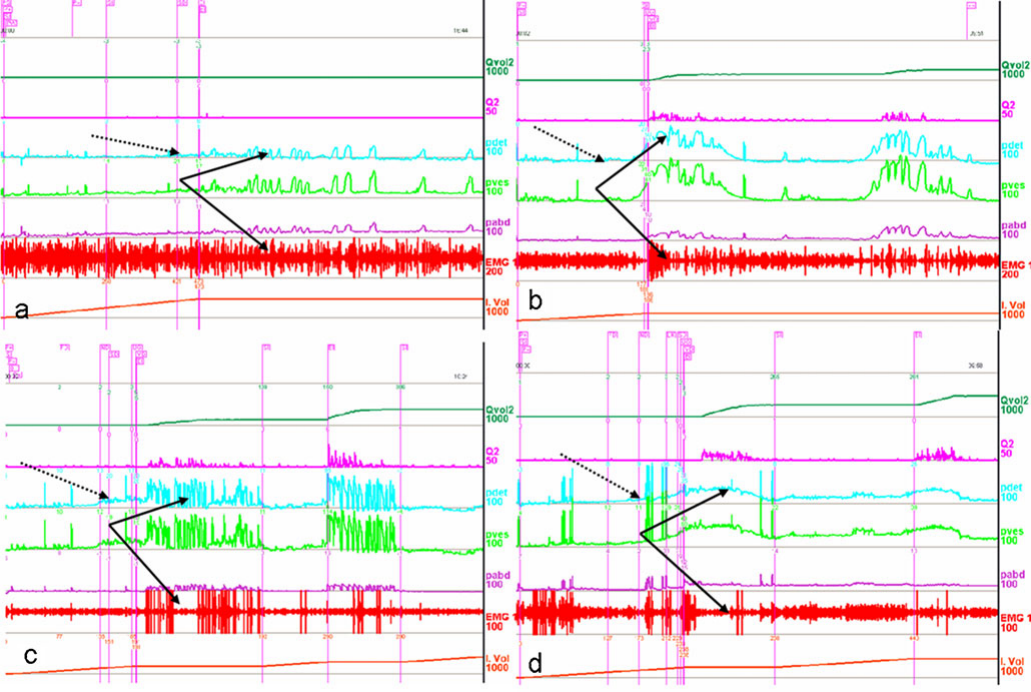
Comprehensive urodynamic tracings of female patients with detrusor underactivity and normal compliance and the efficacy of LFE are displayed. The *dashed arrows* indicate normal compliance and *solid arrows* indicate the state of the detrusor contractility and the sphincter relaxing ability during the voiding phase. **a** A 32-year-old woman with detrusor underactivity. **b** After LFE, her detrusor function recovered, sphincter overactivity improved and she dispensed with catheterization thereafter. **c** Urodynamic study of a 52-year-old female patient showed that the detrusor was underactive and the sphincter overactive before LFE. **d** After the procedure, the detrusor became contractile and sphincter overactivity still remained

The fashion of detrusor contraction is parabola in detrusor intact or fully regained, wavelike in detrusor still weak and not fully regained, and low or high sustained in detrusor unresponsive to treatment. Of the 28 patients in the LFE-NC group, 23 (82 %) regained detrusor contractility after LFE. The pattern of their detrusor contraction changed from low sustained contraction (in 8 cases) to normal parabola contraction (Fig. 3a, b), or from wavelike contraction (16 cases) to normal parabola contraction (Fig. 3c, d). Of the 25 CON-NC patients, only 2 had her pattern changed from low sustained contraction to parabola contraction. None of those of LFE-LC or CON-LC patients regained normal detrusor contractility. The per cent of patients in the LFE-NC group who relied on catheterization for bladder emptying decreased by 43 % (from 54 to 11 %, *p* < 0.01). Those of the LFE-LC and CON-NC groups decreased by only 4 or 12 % (from 85 or 60 % to 81 or 48 %, respectively, *p* > 0.05); those of the CON-LC group decreased by zero and none regained detrusor contractility.

**Fig. 4:**
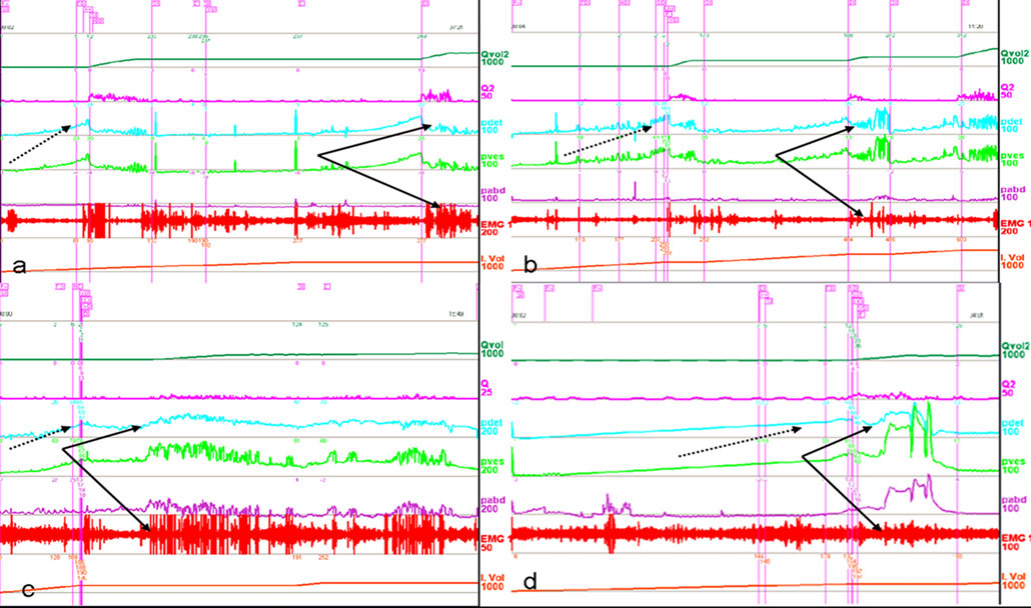
Comprehensive urodynamic tracings of female patients with detrusor underactivity and low compliance and the failure of LFE are displayed. The *dashed arrows* indicate lower compliance and *solid arrows* indicate the state of the detrusor contractility and the sphincter relaxing ability during the voiding phase. **a** A 38-year-old woman was found to be DUA and LC, urinating by abdominal straining and relying on catheterization for bladder emptying. **b** After LFE, her detrusor contraction remained poor in spite of slight increase of the detrusor contractility and she still relied on catheterization. **c** A 54-year-old female patient complained of voiding difficulty with a possible cause of intervertebral disk hernia; urodynamic study showed DUA and LC. **d** After the LFE procedure, her detrusor and sphincter state remained without improvement at all. As an improvement, her abdominal straining in a parabola fashion was more powerful than before

Apart from the slight increase in the detrusor pressure at the end of follow-up study in one case, DUA persisted in patients in the LFE-LC or CON-LC groups, showing low compliance, abdominal straining and sphincter overactivity (Fig. 4a, b), and they still relied on catheterization for bladder emptying except for two patients, whose square-shaped abdominal straining during the voiding phase compensated well for the detrusor and nearly replaced detrusor contraction with full emptying of the bladder (Fig. 4c, d). The study indicated that LFE functioned well for DUA patients with normal compliance but less than satisfactory for DUA patients with low compliance.

## Discussion

Various methods have been accepted to treat DUA in association with a slight degree of bladder outlet obstruction so far [14, 15]. For DUA without obvious mechanical factors involved, effective measures are still lacking. Physical therapy may afford an option for this kind of DUA, especially at the early stage of the disease. One of the major issues in electrotherapy relates to the decision-making process regarding questions such as which modality should be used and with which treatment parameters. LFE applied to the detrusor may cause strengthening of the detrusor by promoting the local blood vessel dilation, increasing blood circulation of the bladder and improving regional alimentation. The electrical current passing through the tissues forces nerves to depolarize, and thereby causes the nerves to “fire” [16]. LFE may stimulate the detrusor along with its neighbouring fascia and connective tissue to contract and relax rhythmically [17, 18]. DUA due to neuromuscular deficiency may require a relatively long period to regain its normal contraction. In the early stage of DUA, the process may be reversible with a drastic intervention. If the DUA process enters into an irreversible stage, the therapy may fail to recover the detrusor function. It is likely that it reaches an irreversible stage, if low compliance occurs. Therefore, it is recommended that active intervention be implemented for patients with DUA at an early stage.

However, in women with a normal relaxation of the pelvic floor, little or no detrusor contraction is needed for complete voiding. These patients are considered to be “normal” [19]. This is an occult modality and should be considered as asymptomatic DUA. The natural history of detrusor dysfunction, either overactive or underactive, cannot be defined simply by symptomatic evaluation. Based on the complaints of the patients only, or even on guidelines issued by authoritative organizations, the findings may still have higher false-positive rates [20, 21]. DUA must be defined by comprehensive urodynamic evaluations, especially the PFS and EMG results [22]. Likewise, the fact that detrusor contraction has been regained should also be validated by a scientific procedure.

There are neurogenic or non-neurogenic factors responsible for DUA. According to the European Association of Urology (EAU) guideline on neurogenic LUTD, this kind of DUA (detrusor underactive/sphincter overactive or normal active) may have an underlying aetiology involving a lumbosacral lesion [1]. As no obvious lesions were found in some patients at present, the dysfunction may be considered as of non-neurogenic origin. We think it is likely that an intermediate zone exists between neurogenic and non-neurogenic LUTD. As far as the functional analysis is concerned, pertaining to either neurogenic or non-neurogenic LUTD, the management guideline for them remains the same.

Results of bladder emptying involve a balance between detrusor muscle contraction and bladder outlet resistance. Clinically, DUA is usually underestimated and has a high prevalence in the older members of the population. Even without overt neurological diseases, the aging process may result in modest declines in detrusor contractility [7, 23]. DUA might be supposed as a result of aging of the detrusor. Such a subject warrants further research in the future.

For those patients, LFE and other conservative procedures failed to initiate contraction of the detrusor, and surgical intervention may be an option. With the advent of minimally invasive approaches, lower urinary tract reconstruction has been reported with increasing frequency [24]. Another treatment for neurogenic DUA is the newly built reflex for rehabilitation of the bladder function [25–28]. Upon completion of the procedure, detrusor contraction and satisfactory voiding were initiated voluntarily by scratching the skin at the appropriate site [22, 25–28].

It should be pointed out that the present study has several limitations. First, our study was performed in one institution and was not a multi-centre study. Second, the sample size of patients was relatively small, and the patient treatment selection was mainly based on their desires. Third, we have not compared the present data with those collected before LFE was introduced in this institution, so that a longitudinal population-based survey was not available [28]. We could conclude that LFE was more effective for female patients with DUA-NC than with DUA-LC. Continued research into its intrinsic mechanism in this field is necessary in order to achieve the maximum benefit for patients with LUTS.

## Conclusions

LFE was more effective for female patients with DUA-NC than those with DUA-LC. Female patients with DUA-NC due to neuromuscular deficiency at its early stage benefited from LFE but those with DUA-LC did not benefit from it.
